# Supplementary material for: Are carbon emissions trading and green financial instruments synergistic? -Comprehensive quantitative research based on content analysis
Source: PLoS One. 2024 Mar 7;19(3):e0298601. doi: 10.1371/journal.pone.0298601 (PMC10919601; doi:10.1371/journal.pone.0298601)
Supplement: S1 Data — (ZIP) [file pone.0298601.s001.zip › Data/地级市Z_F工作报告.zip!/║╙─╧/╓ú╓▌╩╨/└┤╫╘╣1⁄2╓┌║┼í╛┬φ┐╦╩2╛▌═°í┐_2005.docx]

各位代表：

　　现在，我代表市人民政府，向大会作工作报告，请予审议，并请各位政协委员和列席人员提出意见。

　　一、2004年工作回顾

　　过去的一年，全市人民在市委领导下，以邓小平理论和“三个代表”重要思想为指导，认真贯彻党的十六大和十六届三中、四中全会精神，坚持科学发展观，积极落实中央宏观调控政策，切实抓好“五个一百”工程，全市经济发展实现了速度与质量、效益的统一，各项社会事业协调发展，较好地完成了市十二届人大一次会议确定的各项任务。

　　初步统计，全年实现生产总值1375亿元，增长15.5%，是亚洲金融危机以来增速最高的一年，人均生产总值达到2350美元。地方财政收入114.8亿元，增长32.7%；金融机构年末存款余额达到2724.8亿元，增长11.9 %；非公有制经济完成增加值687亿元，增长25%，占全市生产总值的比重达到50%。完成或超额完成了新增就业再就业10万人、工业技改投资100亿元、非公有制经济增加值新增100亿元、地方财政收入100亿元、新设立外资企业100家、规模以上工业销售收入突破1000亿元的“六个一”目标。全市经济进入全面提速、加快转型的新阶段。

　　(一)农业生产稳定增长，农村经济全面发展。认真贯彻落实“中央一号”文件，“三农”工作取得了明显成效。农林牧渔业完成增加值61亿元，增长5.7%。农民收入显著增加，粮食等农产品产量全面增长，农业现代化水平进一步提高。新建奶牛养殖小区20个，新增无公害农产品基地20万亩，农业综合机械化水平达到53%。农业产业化经营步伐加快，休闲观光农业发展迅速。农村税费体制改革成果得到巩固，农民负担大幅减轻。全市所有贫困村和市辖各区、巩义市免征农业税，其余县(市)农业税税率降低3个百分点，农民人均较上年减负81%。扶贫开发工作取得新成效，全市又有1.5万贫困人口脱贫。改建、新建农村公路297.4公里。建成集雨水窖1.01万个，解决了6万人饮水困难。完成沿黄风沙源生态治理造林16.7万亩，生态环境有所改善。

　　(二)工业经济快速发展，经济效益大幅提高。全市规模以上工业完成增加值402.7亿元，增长23.7%；实现销售收入1189.6亿元，增长35%；实现利润74.5亿元，增长38.7%；工业经济效益综合指数达到160.2%，提高16.7个百分点。汽车、煤电铝、装备制造业、食品等优势行业完成增加值272.4亿元，占规模以上工业增加值的67.6%。重点技改项目投资突破百亿，技改投入连续3年实现翻番。全年新增电力装机容量99.6万千瓦、铝加工能力20万吨、卷烟2.7万大箱。大企业大集团规模日益壮大，有50户企业销售收入在3亿元以上，宇通集团、中铝河南分公司突破50亿元。一批高技术企业快速发展，技术创新效果显著，信息化带动作用突出，品牌效应不断增强，企业核心竞争力大幅提高。以产权制度改革为重点的国有工业企业改革取得新成果，提前完成企业中小学移交任务；郑州日产和东风股份成功重组，金星集团与百威公司、奥克公司与生力集团合作进入实质性操作。

　　()消费市场繁荣稳定，新型业态发展迅速。全市社会消费品零售总额558.7亿元，增长16.4%，其中，批零贸易和餐饮业分别完成459.9亿元和80.2亿元，增长13.6%和38.5%。零售业综合竞争力明显增强，天津家世界、德国麦德隆等知名零售企业入驻郑州。圃田建材、东风路科技等交易园区持续发展。开工建设商品房470万平方米，增长34.3%。香江、澳柯玛等物流园区建设进展顺利。会展经济逐步推行市场化运作，成功举办了第十届全国商品交易会。旅游景区建设取得重大进展，旅游总收入达到138.5亿元，增长32%。国有商贸企业改革稳步推进。

　　(四)固定资产投资持续增长，薄弱环节得到加强。积极贯彻中央宏观调控政策，对在建、拟建项目进行了全面清理。克服不利因素影响，全社会固定资产投资完成650亿元，增长30%。重点工程建设继续保持增长势头，郑州出口加工区标准厂房、农业路穿铁路编组站立交、107国道辅道等项目建成投入使用；郑煤白坪矿井、正大世纪城市广场等项目按计划推进；郑州燃气调峰电站2×35万千瓦机组、郑东新区热电厂2×20万千瓦机组、郑州铝业铝板带箔一期、宇通汽车零部件工业园等项目开工建设。加大薄弱环节的投入力度，交通、环保、农林水、社会事业、旅游等方面的基础设施建设得到加强。

　　(五)城市建设取得新进展，城市化进程不断加快。中心城区保护改造进展顺利。续建、新修道路28条，改造支路背街小巷126条、积水点49处，改造、新建雨污水管网25.6公里。完成340余幢楼体夜景照明。全年新增绿地面积510万平方米，新建游园34个。熊耳河滨河公园建成开放，东风渠两岸景观绿化即将完工。郑花路、西北环道、科学大道整治绿化任务基本完成，桐柏路、沁河路等30条路段新植了行道树。继续实行“两级政府、三级管理、四级网络”管理体制，城市管理长效机制进一步完善，市容环境不断优化，空气质量二级以上天数达到81.4%。

　　郑东新区建设全年完成投资60.2亿元。中央商务区形象初现，内、外环60栋高层项目中已有35栋开工建设，其中19栋结顶，楼市销售良好。郑州国际会展中心土建工程基本完工，河南艺术中心地下工程已经结束，中央商务区中心湖破土动工。起步区内基础设施建设基本完成，“三河一渠”绿化工程进展顺利。招商引资成效显著，全年引进项目50个；烟草集团、天津顺驰、上海绿地、澳门宝龙等知名企业纷纷入驻。龙湖南区、商住物流区项目进展顺利。龙子湖区6所高校开工建设。

　　卫星城市及重点镇建设速度加快。卫星城市规划水平提升，投入力度加大，小城镇建设日趋规范。竹林镇等9个乡镇被确定为国家级重点镇。全市城市化率57.9%。

　　(六)对外开放步伐加快，利用外资水平有所提高。新设立外商投资企业117家，实际利用外商直接投资2.5亿美元，增长61.6%；出口创汇10.8亿美元，增长23.8%。成功举办首届世界传统武术节，签订对外合作项目20项，合同利用外资3.9亿美元、域外资金29.8亿元人民币。创新招商方式，市场化、专业化招商迈出了可喜步伐。以非洲为重点，进一步开拓国外市场。出口加工区封关运行，高新技术产业开发区、经济技术开发区招商工作取得新成效。区域经济交流与合作明显加强。

　　(七)就业再就业和社会保障工作成效显著，人民生活明显改善。进一步落实就业再就业政策，减免税费3498万元，支出补贴4256万元，发放国有企业下岗职工生活保障金1.08亿元。实现12.15万城镇求职人员就业再就业，转移农村劳动力就业13.2万人。社会保险覆盖面扩大，社会保险体系进一步完善。养老金按时足额发放率和社会化发放率继续保持100%。全市清理拖欠农民工工资1.46亿元。城市居民最低生活保障标准进一步提高，做到了动态管理下的应保尽保；对符合条件的农村特困户全部实行救助。完善了城市低保户和农村特困户在住房、教育、医疗等方面的配套政策。城市流浪乞讨人员救助管理工作效果明显，与联合国儿童基金会合作开展的流浪少年儿童救助项目，被民政部誉为“郑州模式”。

　　城乡居民收入稳步提高，群众生活质量进一步改善。城镇居民人均可支配收入达到9667元，增长11.8%；农民人均纯收入达到4183元，增长15.2%，首次超过城镇居民收入增速；城市居民人均住房面积达到22.6平方米，农村人均住房面积达到42.7平方米。免费向社会公众开放公益性文化场馆。向社会承诺办好的“十件实事”，除西流湖公园建设缓建外，其余件件得到落实。经济适用房竣工43万平方米，有398户“双困”家庭入住廉租住房。

　　(八)科技教育不断发展，社会事业全面进步。围绕高新技术产业化、制造业信息化，优化配置科技资源，突出科技引导带动作用，荣获“全国科技进步先进市”。教育投入不断加大，市区4所新建高中开工建设，全国第九届中学生运动会体育场馆开始施工，改造农村中小学危房22.7万平方米。加强商城遗址的保护，光荣跻身“中国八大古都”，并加入“世界历史都市联盟”。文化事业进一步繁荣，现代豫剧《嵩山长霞》引起了社会的强烈反响。郑州歌舞剧院顺利组建，创演的《风中少林》得到好评。全民健身运动深入开展。竞技体育取得新成绩，我市运动员孙甜甜荣获第二十八届奥运会网球双打冠军，实现我国网球运动历史性突破；残奥会获得7枚金牌。疾病控制和医疗救治体系建设进展顺利，覆盖城乡的网络初步形成。加强对艾滋病的监测和预防，重大传染病救治工作有序开展。卫生服务设施建设加快，基层卫生条件得到改善。经济普查工作顺利推进，“信用郑州”建设取得新进展。精神文明建设典型层出不穷，涌现出了任长霞、吴玲等先进人物。

　　人口和计划生育工作整体水平进一步提高，基层基础工作得到巩固，政策外生育率和出生二孩率明显下降，流动人口计生工作经验在全国推广。大力整治违法排污企业，建设项目环境管理和自然生态保护工作得到加强。市医疗卫生废物集中处理中心投入使用。整顿土地市场秩序，闲置土地处理工作取得进展。国防教育、民兵、征兵、优抚安置工作顺利开展，军政军民关系更加密切。

　　新闻出版、广播电视、人事、审计、统计、物价、侨务、对台事务、民族宗教、社会科学、地方志、档案管理、气象和地震监测、人防等工作都取得了新的成绩。

　　(九)民主与法制建设得到加强，社会大局基本稳定。自觉接受人民代表大会及其常委会监督，接受人民政协民主监督，认真听取民主党派、工商联、无党派人士、各人民团体的意见。全年办复人大代表议案、建议和政协委员提案1180件。扩大政务公开范围，政府新闻发言人制度开始实施，政府决策的科学性和透明度提高。坚持依法治市，提请通过地方性法规12件，制订规章8件。贯彻执行《行政许可法》，停止执行68项行政许可项目。加强对规范性文件合法性审查，依法规范政府行政行为。

　　切实做好信访突出问题及群体性事件集中处理工作。全市抽调2200余名机关干部，深入开展不稳定因素排查调处，处理了900余起多年积压的信访案件，集体上访上升势头得到遏制，群体性事件妥善处置，信访秩序明显好转。严密侦控、严厉打击境内外敌对势力、敌对分子和“法轮功”等邪教组织的破坏活动。强化综合治理，社会治安保持稳定。加强干警队伍建设，完善快速反应机制，以快制快，震慑犯罪。落实安全生产责任制，加强了安全监管网络和应急救援体系建设。开展经常性的安全检查，加大事故隐患整改力度，全市安全生产事故总量大幅度下降。

　　各位代表，过去的一年，我市经济社会发展成绩的取得，是省委、省政府和市委正确领导的结果，是市人大、市政协支持和监督的结果，是全市人民团结奋斗的结果。在此，我代表市人民政府，向给予政府工作大力支持的人大代表和政协委员，向全市广大工人、农民、知识分子和干部，向驻郑人民解放军、武警官兵、公安政法干警，向各民主党派、工商联、人民团体和各界人士，表示崇高的敬意！向关心和支持郑州发展的港澳台同胞、海外侨胞和国际友人，表示衷心的感谢！

　　在看到成绩的同时，也要看到经济社会发展中一些不容忽视的困难和问题。一是融资困难、运力紧张、建设用地不足等制约我市经济发展的瓶颈问题更加突出。二是安全生产形势依然严峻，重特大事故时有发生。三是群众关注的热点难点问题还没有得到根本解决，群体性事件偶有发生。四是对外开放水平不高，利用外资规模较小。五是城乡居民收入还有待进一步提高，不少低收入群众生活还比较困难。六是就业岗位不能充分满足需求，就业压力仍然较大。七是一些政府工作人员服务意识不强、效率不高，政府廉政建设有待继续加强。这些问题需要在今后的工作中切实加以改进和解决。

　　二、2005年政府工作的总体要求和预期目标

　　2005年是“十五”计划的最后一年，也是进一步巩固和发展宏观调控成果、保持经济社会发展良好态势的关键一年。做好今年的各项工作，对于全面完成“十五”计划，为“十一五”规划实施奠定良好基础具有十分重要的意义。当前，我们正处在重要的发展战略机遇期。从国际环境看，经济全球化趋势继续发展，世界经济仍处于复苏增长期，发达国家产业向我国转移步伐加快，并由沿海向内地延伸，为我市吸引国外资金、承接产业转移提供了难得的机遇；随着今年世贸组织纺织品配额取消，我国与东盟之间的货物自由贸易开始启动，扩大出口将迎来新的机遇。从国内形势看，我国经济仍处于新一轮增长周期的上升阶段，国家提出并实施促进中部地区崛起、加快区域协调发展的战略，今年中央将继续加强和改善宏观调控、巩固宏观调控成果，这些都为我市经济平稳快速发展提供了良好的环境。省委、省政府实施“中原城市群”发展战略，为我市加快发展、提高首位度提供了契机。今年南水北调中线穿黄工程、郑州至西安客运专线等重大项目开工建设，一大批交通、能源、农林水、生态环保、社会事业项目陆续启动，投资规模继续增长，投资对经济的拉动效应将继续显现。我市经济综合实力明显增强，为加快发展奠定了坚实基础。面对新的国际国内形势，面对周边城市你追我赶的竞争态势，我们必须不断解思想，开拓创新，扬长避短，发挥优势，努力推动各项工作再上新台阶。

　　今年政府工作总体要求是：以邓小平理论和“三个代表”重要思想为指导，认真贯彻党的十六大和十六届三中、四中全会精神，坚持以人为本的科学发展观，继续贯彻落实宏观调控政策；解放思想，创新机制，深化改革，扩大开放；调整结构，加快发展，保持稳定，为民谋利；努力建设生态郑州、信用郑州、和谐郑州，促进经济社会全面协调和可持续发展。

　　经济和社会发展的主要预期目标是：全市生产总值增长13%；地方财政收入增长14%；全社会固定资产投资增长25%；社会消费品零售总额增长13%；外贸出口增长25%，实际利用外资增长30%；居民消费品价格总水平上涨控制在4%左右；城镇居民人均可支配收入增长8%，农民人均纯收入增长7%；人口自然增长率控制在6‰以内；城镇登记失业率控制在4%以下。

　　实现上述预期目标，必须从省会的实际出发，牢牢把握以下几点：

　　第一，必须认真贯彻中央加强和改善宏观调控的各项政策措施，充分体现区别对待、有保有压的原则，适度加大关系全局的重大项目和薄弱环节投入力度，增强投资对经济增长的拉动作用。突出结构调整主线，促进传统产业的转型升级，努力实现经济发展速度更快、结构更优、质量更高、效益更好。

　　第二，必须坚持以人为本的原则，切实加强就业再就业和社会保障工作，认真落实促进农民增收的政策措施，加大扶贫开发力度，千方百计增加城乡居民收入，培育新的消费增长点，增强消费对经济增长的拉动作用。

　　第三，必须按照标本兼治的原则，推进体制创新，深化关键领域的改革。认真解决经济运行中存在的突出问题，更加注重解决体制性、机制性深层次矛盾，为经济持续快速发展注入活力。

　　第四，必须进一步解放思想，不断扩大利用外资规模，提高对外开放水平。不断创新招商引资方式，大力提高利用外资质量，推动产业升级和技术创新。加快外贸增长方式转变，推进贸易方式和市场的多元化。

　　第五，必须统筹城乡、区域和经济社会协调发展，促进人与自然的和谐。努力打破城乡二元结构，形成合理的产业布局，加快工业化、城市化进程。高度重视可持续发展，把控制人口规模、节约资源和保护环境摆在突出位置，努力实现经济效益、社会效益和生态效益相统一。

　　第六，必须全力实现好、维护好、发展好人民群众的根本利益。牢固树立“群众利益无小事”的观念，把关心和服务群众作为第一职责，把群众的评价作为衡量工作政绩的第一尺度，统筹兼顾，正确处理局部利益和整体利益、眼前利益和长远利益的关系。在推进城市化过程中，维护好农民利益；在推进企业改革过程中，维护好职工利益；在推进城市建设过程中，维护好居民利益。高度关注困难群体，着力解决涉及群众切身利益的突出问题，使广大群众在改革与发展中得到更多的实惠。

　　三、加快发展，全面完成“十五”计划

　　在当前区域间、城市间竞争日趋激烈的情况下，我们要增强加快发展的紧迫感和使命感，再鼓干劲，扎实工作，确保全年目标任务和“十五”计划圆满完成。

　　(一)加快经济结构调整和增长方式转变，确保工业经济快速发展。紧紧围绕我市确立的今后4年实现规模以上工业增加值、装备制造业销售收入、工业累计投入“三个一千亿”的发展目标，加大结构调整力度，增强工业发展后劲。积极开展“工业项目建设年”活动，明确责任，定期督查，确保全市工业项目顺利推进，工业投入增长20%以上。突出抓好中铝河南分公司70万吨氧化铝、郑煤集团赵家寨300万吨煤矿等一批重点项目。加大对重点企业的扶持力度，集中支持宇通、日产、中铝河南分公司、烟草集团、三全等50户重点企业发展；继续实施名牌战略，努力培育一批核心竞争力强、在国际国内市场占有率高的大企业大集团。力争实现销售收入超百亿元企业1家，超50亿元企业2家，超10亿元企业20家以上。继续加大技术改造投入力度，运用高新技术、先进适用技术改造传统产业。在抓好煤电铝、装备制造业、食品等行业的同时，发展高新技术产业，加大电子信息、生物化工与医药、新材料等行业的发展。坚持不懈地推进工业企业信息化，提高工业领域现代化水平和管理效率。

　　(二)加强“三农”工作，推动农村经济持续发展。坚持“多予、少取、放活”的方针，加大对“三农”的扶持力度，以城市反哺农村，以工业反哺农业。继续推进农业与农村经济结构战略性调整，按照都市型、近郊型和远郊型农业圈层的划分进行农业区域布局调整。抓好现代农业示范园区建设，推进农业产业化经营。完善农产品质检体系。加大农业基础设施投入，改善农业生产和农民生活条件。全面启动森林生态城市建设，全市新增造林面积22.7万亩。制订水资源利用总体规划，发展节水型农业，重点抓好一批节水灌溉工程建设，解决好缺水地区安全饮水问题。继续加大对农村各项事业投入，统筹城乡经济协调发展。继续实行最严格的耕地保护政策，稳步推进农村土地承包经营权流转。进一步减轻农民负担，继续落实粮食直补政策，在全市免征农业税。搞好第五届村委会换届选举工作，抓好村务管理和民主管理，保持农村社会稳定。

　　(三)充分发挥商贸优势，大力发展现代服务业。进一步加强城区交易市场建设，利用现代信息手段提升批零贸易层次，加快农贸市场改造升级，逐步形成层次分明、便利市民的市场网络。加大国内外知名商贸企业引进力度，发展连锁经营等新型业态。积极利用外资加快流通企业的改组改造，充分发挥郑州铁路、公路、邮政、航空区位优势，搞好物流规划，整合物流资源，提高信息化水平，逐步形成具有郑州特色的现代大物流体系。依托郑州国际会展中心，承办大规模、高规格的会展活动，努力培育有特色的会展品牌。继续深化住房制度改革，大力发展、完善、规范房地产市场，切实加强对经济适用住房开发建设与销售的管理。整合旅游资源，搞好重点景区的策划工作，完成主要景区环境整治，加大旅游宣传促销力度，规范市场秩序，提升旅游业整体形象和服务水平。围绕建设区域性金融中心城市的目标，做好规划，制定方案，重视和支持金融、证券、信托、期货市场的培育，创新体制，搞活机制，做大做强金融企业。同时积极创造条件，引进外资金融企业，开发新的金融产品，组织更多的资金支持经济社会发展。积极扶持中介、咨询、信息等服务业，推动我市现代服务业快速发展。

　　(四)积极推进城市建设和城市化进程，促进城乡区域协调发展。以创建国家园林城市、国家卫生城市、国家环保模范城市、全国文明城市为主线，加强中心城区保护改造，确保“三年大变”目标的实现。重点对107国道(黄河路立交?陇海铁路立交)进行综合整治，对金水路东段(107国道?东明路)实施升级改造，打通未来大道金水路至航海路段，完成东风渠综合整治，使之成为郑州市的靓点。抓好商城遗址公园、月季公园等一批公园建设，建成区绿化覆盖率达到35%。继续实施路灯和夜景照明工程。举办首届中国月季展览会，再现市花风采。加大基础设施建设力度，开工建设北三环?文化路立交、西三环?化工路立交、建设路?解放路立交等7座立交桥；续建、新建冉屯路、迎宾路等18条市政道路。重点抓好大气、水、噪音污染治理，改善市区环境空气质量。抓好背街小巷改造、市容街景整治、公园游园建设、环境卫生和环卫设施建设等。坚持建管并重，不断提高城市管理水平。加大市容环境整治力度，重点整治户外广告和小饭店、小理发店、小熟食店、小副食店、小冷饮店，坚决取缔占道经营、露天烧烤和燃煤大灶。加强地名管理，规范地名标牌设置。

　　按照“三年出形象”的要求，突出重点，加大投入，强力推进郑东新区建设。加大融资力度，拓宽融资渠道，积极争取金融与投资机构的支持。加大重点项目推进力度，中央商务区内外环高层开工总数达到45栋以上，会展中心竣工交付使用，省艺术中心主体工程、中央商务区中心湖基本完成，会展宾馆力争开工。通过上述项目的实施，使中央商务区的景观初步展现，市政设施配套到位，基本具备办公和开展商务活动的条件。龙湖南区建设全面推进，商住物流区项目基本摆满，龙子湖高校区3所高校具备教学条件。

　　高起点完成城镇体系规划修订工作，不断加大规划引导和政策扶持力度，加快卫星城市和小城镇建设，推进城市化进程。重点加强城镇供水、污水和垃圾处理等基础设施建设，保护生态环境，提高公共服务水平。规划建设好6个县(市)城区，建设各具特色的卫星城市；注重把城镇化与工业化结合起来，加快20个重点镇的规划建设，形成以郑州中心城区为核心、各卫星城和中心镇众星拱月、有序发展的格局。

　　(五)强力推进各项改革，不断扩大对外开放。鼓励外资、市外优势企业及市内民营企业参与重点国有企业的改组改造，基本完成国有工业和商业企业改制任务。推进粮食流通体制改革，全面放开粮食购销市场，完成国有及国有控股参股粮食购销企业改组改造。加快农村信用社改革，改善农村金融服务，加大金融支农力度。继续推进农村税费改革，规范涉农收费。深入开展投融资体制改革，推行项目核准制和备案制管理，做好代建制试点工作，放宽社会资本投资领域。积极推进社会事业和公用事业体制改革。深化事业单位体制和人事制度改革，继续实施人才强市战略，加快培养、积极引进各类人才，为我市经济发展提供智力和人才保障。

　　进一步解放思想，更新观念，下大气力优化投资环境，落实招商引资各项优惠政策。创新招商方式，实行专业招商、定向招商、以商招商等，千方百计吸引跨国零售商业巨头、大型物流企业和高科技产品生产企业落户我市。加大教育、文化、旅游、医疗卫生、中介服务等领域的招商力度。做好利用国外贷款工作。继续实施东引西进战略，有针对性地吸引东部地区的产业和资本向我市转移。组织多层次、宽领域的市情和项目推介会，争取更多的项目和资金入驻郑州。大力推广“大通关”制度和“电子口”联网建设，建立和完善快速通关机制。突出发展重点，加大招商力度，郑东新区、高新技术产业开发区、经济技术开发区、出口加工区要充分发挥区位和政策优势，努力吸引境内外资金。

　　(六)加快非公有制经济和县域经济发展，提升整体经济发展水平。完善政策措施，营造良好环境，推动全市非公有制经济发展再上台阶。全面落实促进非公有制经济发展的引导扶持政策，进一步放宽市场准入，健全服务体系。力争非公有制经济增长20%以上，不断提高占全市生产总值的比重。

　　认真贯彻落实中央、省支持县域经济发展的各项政策措施，继续加大对5个扩权市发展的支持力度。充分发挥各县(市)比较优势，积极争取和用好省政府县域经济发展专项资金，重点支持县(市)基础设施和特色工业项目建设。切实加强城乡市场体系建设，抓好使用国债资金的市场和流通项目建设。完善县域经济发展激励机制，抓住省启动豫东平原县域经济快速发展的战略契机，支持中牟县加快发展。

　　(七)切实做好财税金融工作，充分发挥财政金融对经济社会发展的支持作用。突出财政支持重点，增加财政涉农投入，支持解决“三农”问题；实施工业重点投入项目奖励贴息，加快工业经济发展；发挥中小企业信用担保机构作用，支持和促进中小企业发展；落实有关财税扶持政策，加快非公有制和外向型经济发展；加大筹资力度，拓宽城市建设投融资渠道，确保城建规划项目的资金需要。坚持依法治税，强化征管稽查。加强银企沟通与合作，争取金融机构不断增加贷款投放。加大上市公司融资力度，扩大企业融资。规范发展各类消费信贷业务，增强对消费需求的信贷支持。

　　(八)加快发展各项社会事业，促进社会全面进步。加大科技投入，完善科技成果转化机制。实施科技创新、制造业信息化、农村科技富民三大工程。加大教育基础设施投入，新建一批中小学校，改善办学条件；推进市区中学普职分设工作；加快中州大学和郑州师专合并升本步伐；完成市区中学3个体育场和6个体育馆的建设，确保全国第九届中学生运动会顺利举行；继续实施“农村中小学危房改造工程”；整合电教资源，促进现代远程教育。继续推进商城遗址保护及环境整治，注重对古都文化的发掘与研究，加大郑州古都的宣传力度。深化城镇医疗卫生体制改革，加强医疗市场监管；继续完善疾病预防控制和医疗救治体系建设。大力发展慈善事业。进一步做好殡葬改革工作。

　　切实做好人口和计划生育工作，加快建立利益导向机制，加强计划生育基层基础工作，抓好出生人口性别比控制和流动人口计划生育管理服务工作，稳定低生育水平。发展体育事业，深入开展群众性体育活动。加强老龄工作，解决好离退休人员生活、就医等问题。

　　继续做好审计、统计、民族、宗教、气象、人防、地震、档案、侨务、民兵等各项工作。

　　四、保持稳定，努力构建和谐社会

　　坚持以人为本，正确处理改革、发展、稳定的关系，高度重视精神文明建设和社会稳定工作，为我市加快发展创造和谐的社会环境。

　　(一)加强社会主义精神文明建设，努力提高社会文明程度和公民整体素质。以创建“全国文明城市”为目标，深入开展群众性精神文明创建活动。广泛开展社会公德、职业道德和家庭美德教育，继续开展“道德规范进万家”活动，在全社会形成良好的道德规范。深入进行爱国主义、集体主义和社会主义教育，大力弘扬时代精神和民族精神。高度重视青少年思想道德教育，引导青少年健康成长。加强民族理论和政策教育，促进各民族的团结和进步。加强科普教育宣传，破除封建迷信，倡导科学精神和文明健康的生活方式。建立健全社会信用体系，深化诚信宣传教育，推动社会信用制度建设。建设学习型社会，努力构建终身教育体系，全面推进素质教育，增强民众的就业能力、创新能力、创业能力。加强国防教育，深入开展双拥活动，再创“全国双拥模范城”。

　　(二)着力抓好社区建设，完善社区服务功能。以创建“全国社区建设示范市”为目标，加大社区建设投入，完善社区功能，优化、美化居民生活环境，逐步改善社区居委会成员的工作、生活条件。进一步理顺市、区、街道、社区的事权关系，强化社区自我管理、自我教育、自我服务、自我监督功能。加强社区服务信息化、网络化建设，整合社区资源，为居民提供就业、家政、医疗、配送、社会保障服务。繁荣社区文化，加强社区治安，规范社区管理，加快建设管理有序、文明祥和的新型社区。

　　(三)认真做好信访工作，努力化解影响社会稳定的各种矛盾和问题。正确认识和处理新形势下人民内部矛盾所反映的新情况、新问题，解决好人民群众普遍关注的热点、难点问题。建立信访稳定工作长效机制，变群众上访为领导下访，落实信访工作领导责任制，严格实施责任追究制。明确各级各部门维护稳定工作的职责任务，完善定期排查、归口调处、领导包案、督查督办制度，加强维护稳定的各项基层基础工作。完善公共安全等突发事件应急处置预案，建立健全反应灵敏、运转高效的应急处置工作体系，提高处置突发公共事件的能力。

　　(四)扎实推进社会治安综合治理，严厉打击各种犯罪。坚持“严打”方针不动摇，始终保持对犯罪分子的高压态势，严厉打击和震慑各类严重刑事犯罪，全面提高现场抓获率、破案率。继续强化社会治安管理服务工作，加强对暂住人口、流动人口、刑释解教人员、吸毒人员、闲散青少年的教育管理。采取有效措施，努力扫除“黄、赌、毒”等社会丑恶现象。重点加强对杂居院、城中村的治安管理和防范工作，继续推广城中村治安防范社区化、出租房屋旅栈化、暂住人口常住化的管理模式，进一步提高城中村流动人口服务管理和治安防范工作水平。深入开展创建安全院、楼、村活动。全面落实社会治安综合治理各项措施，提高治安管理的信息化水平和治安防控能力。

　　(五)切实抓好安全生产，保护人民群众生命财产安全。深入开展以矿山、建筑施工等高危行业为重点的安全生产专项整治，加强道路交通和消防安全管理，坚决取缔、关闭非法和不具备安全生产条件的小矿小厂、经营网点，集中力量整改一批危及人民群众生命财产安全的重大事故隐患。坚持把专项整治与落实生产经营单位安全保障制度，与开展企业安全生产质量标准化活动紧密结合起来，建立安全生产长效机制。加强市、县、乡三级安全生产监管机构建设，健全监督执法体系，严格实行奖惩制度，遏制重特大安全生产事故的发生。加大食品药品市场监管力度，抓好河道治理和大中型水库除险加固，确保人民生命财产安全。

　　五、为民谋利，不断提高人民群众生活水平

　　千方百计为民谋利、维护好群众利益、不断提高群众生活质量和水平，是政府工作的根本所在，是我们加快发展的最终目标。

　　(一)完善城乡就业服务体系，努力扩大就业规模。广开就业渠道，大力开发公益性岗位，重点做好下岗失业人员特别是“4050”人员再就业工作。继续落实就业再就业各项优惠政策，加大再就业资金投入。抓好再就业各项补贴资金的落实，切实简化小额担保贷款手续，加快小额担保贷款的发放。继续做好农村劳动力就业培训工作，运用劳动保障就业信息网络，实现城乡互联、信息共享，提供求职登记、职业指导、职业介绍和劳动保障事务代理等“一站式”服务，不断完善城乡就业服务体系，促进农村劳动力转移就业。

　　(二)健全社会保障制度，搞好城乡社会保障。加大社会保险扩面征缴力度，在职职工参保人数达到59万人。继续扩大医疗保险覆盖面，年底参保人数达到40万人，医疗保险费征缴率达到98%。继续做好失业保险和工伤保险工作。积极推进农村养老、医疗、保险等社会保障体系建设，搞好以大病统筹为主的新型农村合作医疗试点，构筑新型社会保障平台。

　　(三)千方百计增加城乡居民收入，不断改善城乡居民生活。努力改善农村贫困地区的生产生活条件，完成第二批30个重点扶贫村的整村推进任务，继续抓好新密袁庄综合扶贫开发区和巩义、登封的移民搬迁扶贫工作。继续加大对拖欠农民工工资清欠工作力度，清理对农民工的歧视性政策，切实保障农民工合法权益。严禁违法征用农民土地和截占、挪用土地补偿费用。严格实行最低工资制度，认真落实劳动法规定的保障职工收入的各项政策，采取积极措施，增加城镇居民收入。

　　(四)做好社会救助工作，解决困难群众实际生活问题。高度关注城乡困难群体，加大财政支持力度，做好城市低保和农村特困户救助工作，做到分类施保、应保尽保。适当补贴困难企业离退休职工生活费和医疗保险费，适时提高城市最低生活保障线，帮助城市特殊困难家庭解决看病难、上学难、住房难等实际困难。做好城市流浪乞讨人员的救助管理工作。完善农村五保户生活保障制度。建立完善资助贫困学生的政策和制度。进一步完善灾害救助体系，落实配套资金，加强对救灾资金发放的监督与管理，确保灾民基本生活。

　　(五)认真解决群众反映的热点问题，努力办好十件实事。通过广泛征集群众意见，2005年，市政府将集中财力，努力为群众办好以下十件实事：1、全市城镇12万人实现就业再就业，转移农村劳动力12万人，建立专业农民工劳务市场。2、市区新建22所中小学校，解决中小学入学难问题。3、新开工经济适用住房50万平方米，竣工40万平方米，廉租住房覆盖率达到符合条件“双困家庭”100%。4、打通市区断头路9条，新建街头游园30个，改造积水点45处。5、市区新建100座公厕，增加公厕指示标志，解决市民入厕难问题。6、优化、新增公交线路30条，新增公交车300标台。7、投资1500万元，进一步扩大农村合作医疗试点，创建示范乡镇卫生院。8、在农村实施“三个一万”工程，建设沼气池1万个，西部缺水山区新建集雨节灌水窖1万个，贫困地区实现1万人脱贫。9、净化市区大气环境，市区环境空气质量二级以上天数达到75%以上。10、改善城市生态环境，完成森林生态城工程造林10万亩，市区新增绿地500万平方。

　　六、建设服务型政府，努力提高政府工作水平

　　加强政府自身建设，转变政府职能，改进政府工作，努力建设服务型政府，是全面完成各项目标任务的重要保证。

　　(一)完善民主决策机制，提高科学决策水平。各级政府要坚决贯彻人大及其常委会决议，坚持重大事项报告制度，自觉接受人大及其常委会监督。认真接受政协的民主监督，广泛听取各民主党派、工商联、无党派人士、社会团体的意见和建议。完善人民建议征集、重大事项社会公示、社会听证、专家咨询制度，健全政务信息公开制度，提高决策的透明度和公众参与度，保障人民群众的知情权、参与权和监督权。完善重大问题的议事规则和决策程序，加强调研工作，充分听取各方面意见，民主决策、科学决策。

　　(二)加强政府法制建设，全面推进依法行政。继续深入贯彻执行《行政许可法》和《国务院全面推进依法行政实施纲要》，不断提高依法行政的能力，加快建设法治政府。全面推进依法治市，建立权责明确、行为规范、监督有效、保障有力的行政执法体制。加强行政监察和执法监督，严格执法，文明执法，公正执法，提高政府的公信力。健全完善法律援助体系，切实保障弱势群体的合法权益。

　　(三)改进管理方式，提高政府工作效能。正确履行政府职能，按照履行好经济调节、市场监管、社会管理和公共服务四大职能的要求，注重运用经济和法律手段管理经济，真正把政府经济管理职能转变到主要为市场主体服务和创造良好发展环境上来。更加重视履行社会管理和公共服务职能，把财力、物力更多地用于社会管理和公共服务，把精力更多地放在推进社会发展和解决民生问题上。按照上述基本要求，创新行政方式和运行机制，推广电子政务，加快市、县、乡三级政务信息平台建设，逐步开展网上审批。改革行政审批制度，优化行政流程，逐步推进前置审批向后续监管的转变，确保政府工作效能全面提高。切实加强人事编制工作，严格控制机构和人员编制。组织编制好“十一五”规划。

　　(四)切实加强勤政廉政建设，不断提高公务员队伍整体素质。坚持标本兼治、综合治理，惩防并举、注重预防，抓紧建立健全与市场经济体制相适应的教育、制度、监督并重的惩治和预防腐败体系。加强政府系统保持共产党员先进性教育，认真落实廉政建设责任制，以解决群众反映的突出问题为重点，坚决纠正损害群众利益的不正之风。加强廉政制度建设，真正形成用制度规范从政行为、按制度办事、靠制度管人的有效机制，保证公务员廉洁从政。推进公共财政体制建设，完善预算编制和执行制度，努力降低行政成本。加强公务员思想道德和纪律教育，营造广大干部学习氛围，在全市政府机关继续深入开展“争当人民满意的公务员”活动。进一步加大行风评议工作力度。

(五)大兴求真务实之风，狠抓各项工作落实。坚持科学的发展观和正确的政绩观，重实践、说实话、办实事、求实效，坚决反对形式主义、官僚主义和弄虚作假。改革会议制度，大力精简各类会议和文件。严格规范和控制各种检查、评比和达标活动。不断完善领导干部联系基层、联系群众制度，深入基层，深入一线，靠前指挥，解决事关经济社会发展的紧迫问题，解决事关千家万户根本利益的具体问题，持之以恒地抓推进、抓落实、抓成效。严格实行责任追究制，对那些因推诿扯皮、欺上瞒下、掩盖矛盾和问题，导致工作棚架、造成严重后果的，要一查到底，严肃处理。

各位代表，实现加快发展，保持稳定，为民谋利的总体要求，任重而道远。需要我们更加昂扬的干事创业激情，需要我们更加求真务实的工作作风。让我们紧密团结在以胡锦涛同志为总书记的党中央周围，坚持以邓小平理论和“三个代表”重要思想为指导，深入贯彻党的十六大和十六届三中、四中全会精神，在市委的领导下，依靠和带领全市人民，同心同德，克难攻坚，脚踏实地，埋头苦干，努力争取提前实现全面建设小康社会的宏伟目标！
